# Loneliness, Insomnia and Suicidal Behavior among School-Going Adolescents in Western Pacific Island Countries: Role of Violence and Injury

**DOI:** 10.3390/ijerph14070791

**Published:** 2017-07-15

**Authors:** Bimala Sharma, Tae Ho Lee, Eun Woo Nam

**Affiliations:** 1 Yonsei Global Health Center, Yonsei University, Wonju, Gangwon-do 26493, Korea; bimalasharma@gmail.com (B.S.); leetaeh5@naver.com (T.H.L.); 2 Department of Health Administration, Graduate School, Yonsei University, Wonju, Gangwon-do 26493, Korea

**Keywords:** loneliness, insomnia, suicidal ideation, suicide attempt, adolescents, pacific island countries

## Abstract

This study aimed to examine whether being bullied, fighting, and injury, regarded in terms of frequency and nature, were significantly associated with psychological distress and suicidal behavior, independent of substance abuse and parental support in adolescents. Secondary analysis of data from the Global School-based Student Health Survey from Kiribati, the Solomon Islands, and Vanuatu was conducted. Binomial logistic regression analysis was used to examine the association of being bullied, fighting and injury with psychological health outcomes (loneliness, insomnia, suicidal ideation and suicide attempt) at a 5% level of significance. A total of 4122 students were included; 45.5% were male, and 52.0% were 14 years of age or younger. Of the total, 9.3% felt lonely and 9.5% had insomnia most of the time over the last 12 months; 27.6% had suicidal ideation, and 30.9% reported at least one suicide attempt in the last 12 months. Multivariable logistic regression analysis showed that being bullied, fighting and injury were significantly associated with psychological health outcomes; adjusted odds ratios (AORs) of loneliness, insomnia, suicidal ideation and suicide attempt increased with increased exposure to bullying, fighting, and injury compared to non-exposed group. Among the types of bullying victimization, the highest AORs of insomnia and suicide attempt were among students who were left out of activities, compared to the non-bullied. Among the causes of injury, adolescents injured due to a physical attack were the most likely to report the highest AORs of loneliness, insomnia and suicidal ideation compared to those not injured. Preventing violence and injury among adolescents might contribute to better mental health and reduction of suicidal behavior.

## 1. Introduction

Suicidal behavior in adolescents is a common problem in low-income and middle-income countries [1]. Despite the evidence that many deaths are preventable, suicide is too often a low priority for governments and policy-makers [2]. Although World Health Organization (WHO) states that the suicide rate dropped by 47% between 2000 and 2012 in low- and middle-income countries in the Western Pacific Region [2], the literature also reports that suicide was more common in the Western Pacific Region and the Western Pacific Island countries, especially in the countries with faster growing populations, including Papua New Guinea, Kiribati, Vanuatu, and Solomon Islands etc. [3,4]. Loneliness and insomnia are two indicators of psychological distress in adolescents that are significantly associated with suicidal and other health risk behaviors, and poor health outcomes [5,6,7,8]. 

Being bullied and fighting, important issues of violence in adolescents, have been found to be associated with psychological distress including loneliness, insomnia, suicidality, and poor mental and physical health outcomes [6,9,10,11]. In addition, previous studies from different countries have shown that a wide range of socio-environmental, psychological, and behavioral factors have been correlated with psychological distress [5,7,8,12,13] and suicidal behavior in adolescents [5,14,15,16,17,18,19,20]. A study conducted in 32 low- and middle-income countries showed that factors associated with suicidal ideation included experiences of bullying and physical violence, loneliness, limited parental support, and alcohol and tobacco use in adolescents [1]. Loneliness and insomnia, in turn, are reported as significant factors associated with a higher level of suicidal ideation and suicide attempts in school adolescents [1,20]. Moreover, another study also showed that suicide attempters with insomnia more frequently used violent methods compared to other patients [21]. 

Child and adolescent injury is a growing global public health issue, and increases in the age range of 15–19 [22,23]. A higher prevalence of suicidal behavior, bullying victimization and injury was observed in the Western Pacific countries [17]. In addition, unintended injury has been one of the leading causes of mortality among young people in the Region [24]. Injury was found to be associated with bullying and being in physical fights in adolescents [25]. However, there is a shortage of literature examining whether getting an injury is significantly associated with loneliness, insomnia and suicidal behaviors.

The aforementioned studies showed a relationship between being bullied, fighting and psychological distress and suicidal behavior; however, the associations with the frequency and nature of bullying victimization have not been explored. More importantly, there is lack of information about psychological distress and suicidal behavior, and the role of violence and injury in these in the Western Pacific Islands. The two indicators of psychological distress, loneliness and insomnia; and two suicidal behaviors, suicidal ideation and suicide attempt, were assessed in this study. Thus, in the present study, we investigated whether being bullied or bullying victimization, being in fights, and injury, in terms of frequency and nature, were significantly associated with loneliness, insomnia and suicidal behavior independent of substance abuse and parental support in adolescents from three Pacific Island countries. We used the Global School-based Student Health Survey (GSHS) data. The GSHS is a youth risk behavior survey, sponsored by the WHO, conducted primarily in low-and middle-income countries [26]. 

## 2. Methods 

### 2.1. Study Area and Sampling

The Centers for Disease Control and Prevention (CDC) conducted the GSHS in eight Pacific Island countries from 2010 to 2013 and made the data publicly available. The data and details of the GSHS are publicly accessible online (https://www.cdc.gov/gshs/countries/westpacific/index.htm) [27]. The data sets are accessible to the public without revealing any identifiable information of the respondents. In this study, we performed secondary data analysis of the existing data from three Pacific Island countries. The selected countries were Kiribati, Solomon Islands, and Vanuatu; all belong to the lower- middle-income countries group [28]. The surveys were conducted in 2011. For this study, we chose the countries that used a two-stage cluster sampling design and had data on loneliness, insomnia, and suicidal behavior. Thus, Cook Islands and Tuvalu were excluded due to non-use of two-stage cluster sampling design. Samoa was excluded because more than 15% of the data on suicidal behavior was missing; Fiji Islands and Tonga were excluded due to lack of data on suicidal behavior. Data were collected using a two-stage cluster sampling design. In the first stage, schools were selected based on probability proportional to enrollment size. In the second stage, classes from the selected schools were randomly chosen, and all students were eligible to participate. The study response rate was 85% in Kiribati, 85% in Solomon Islands, and 72% in Vanuatu, as reported by the countries in their GSHS survey report. A total of 1582 students from Kiribati, 1421 from the Solomon Islands, and 1119 from Vanuatu participated in the GSHS. Thus, a total of 4122 students were included in the study.

### 2.2. Variable Measurements

The variables were categorized and coded as shown in Table 1. 

### 2.3. Data Analysis 

The data was analyzed using Statistical Package for the Social Sciences (SPSS) version 21.0 (IBM Corp., Armonk, NY, USA). To describe the characteristics of the study population, the proportions were calculated for total sample and individual country. Binomial logistic regression analysis was used to examine if being bullied, fighting and injury were significantly associated with loneliness, insomnia, suicidal ideation and suicide attempt. *p* values < 0.05 and 95% confidence intervals (CIs) were used to identify significance level. Unadjusted and adjusted odds ratios (ORs) with 95% confidence intervals (CIs) and *p* values were computed. An OR greater than 1 indicates that students who were bullied/in fights/injured were more likely to report the negative psychological health outcome (loneliness or insomnia or suicidal ideation or suicide attempt) compared to the not exposed group (non-bullied or never been in a fight or non-injured). The adjusted odds ratio (AOR) was used to measure the strength of association between an exposure and an outcome variable, adjusting with other possible correlates. AOR of loneliness, insomnia, suicidal ideation and suicide attempt for being bullied, fighting and injury were computed separately adjusting with age (as a continuous variable), sex, country, substance abuse and parental support variables. In addition, in a multivariable logistic regression analysis, the ORs of insomnia were also adjusted with loneliness; those of suicidal ideation with loneliness and insomnia, and those of suicidal attempt with loneliness, insomnia and suicidal ideation. The multivariable analysis was also adjusted with the primary sampling unit to account for the cluster sampling design (as a continuous variable). 

## 3. Results

Among the 4122 respondents, 38.4% were from Kiribati, 35.5% from Solomon Islands, and 27.1% from Vanuatu. Of the total respondents, 45.5% were male and 45.9% were 15 years of age or older. Approximately half (49.0%) were bullied at least once in the last 30 days, 44.2% were involved in one or more fights, and 52.4% were injured in the last 12 months. The proportion of students being bullied, involved in a fight, or seriously injured was highest in the Solomon Islands. Among the respondents, 22.5% smoked, 21.4% consumed alcohol in the last 30 days, and 8.4% had used marijuana at least once in their life. Smoking and marijuana use were highest in the Solomon Islands, and alcohol use was highest in Kiribati. Parental guidance and understanding were highest in the Solomon Islands, and parental intrusion on privacy was highest in Kiribati. Among the participants, 9.3% mostly/always felt lonely, and 9.5% had insomnia most of the time/always in the last 12 months. Regarding suicidal behavior, 27.6% seriously considered suicide, and 30.9% attempted suicide at least once in the last 12 months. The prevalence of suicidal ideation was 17.2% in Vanuatu, 29.0% in the Solomon Islands, and 33.6% in Kiribati; and that of suicide attempt was 23.0% in Vanuatu, 36.9% in the Solomon Islands, and 31.0% in Kiribati. The prevalence of loneliness, insomnia, and suicide attempt was highest in the Solomon Islands (Table 2).

Among the respondents, 10% respondents were most kicked, pushed, or shoved, 4.2% were made fun of due to their religion, 5.8% were made fun regarding their sex, and 1.9% of students was left out of activities. Falling was the leading cause of injury (11.4%), followed by something falling and hitting the student (6.9%) and vehicle accidents (3.6%). Overall, 4.3% received a serious injury because they were attacked (Table 3). 

Table 4 shows unadjusted and adjusted ORs of loneliness, insomnia, suicide ideation, and suicide attempts for bullied, fighting, and injury frequency. In both unadjusted and the adjusted models, being bullied 1 to 2 days and ≥3 days was significantly associated with all psychological health outcomes (loneliness, insomnia, suicidal ideation and suicide attempt), and ORs increased with increased exposure to bullying compared to the non-bullied. AORs of loneliness were 1.4 and 1.7 for subjects who were bullied 1 to 2 days and ≥3 days, respectively. Similarly, AORs of insomnia were 1.9 and 2.4, those of suicidal ideation were 2.2 and 2.3, and those of suicide attempts were 2.0 and 2.4 among students who were bullied 1 to 2 days and ≥3 days, respectively. 

Being in fights once, 2 to 3 times or ≥4 times was significantly associated with all psychological health outcomes, and ORs increased with increased frequency of fighting compared to those who had never been in a fight. According to the adjusted model, adolescents who were in fights once, 2–3 times, or ≥4 times were 1.4, 1.9, and 2.4 times more likely to attempt suicide, respectively. Similarly, the AORs of suicidal ideation were 1.5, 1.6, and 1.9 for subjects who were in fights once, 2–3 times or ≥4 times, respectively. 

Sustaining injury once, 2 to 3 times or ≥4 times was significantly associated with all four outcomes, and ORs increased with increased exposure to injury. The AORs of loneliness were 2.0, 2.0, and 3.4 for subjects who were seriously injured once, 2–3 times, or ≥4 times, respectively. Similarly, the AORs of insomnia were 1.9, 2.8, and 2.7 among subjects who were injured once, 2–3 times or ≥4 times, respectively, those of suicidal ideation were 1.5, 1.7, and 2.3, and those of suicide attempt were 1.8, 2.4, and 2.5, respectively. These results show that loneliness, insomnia and suicidal behaviors seem to increase with an increase in the frequency of violence and injury. 

Table 5 shows the association of types of bullying victimization and causes of serious injury with four psychological health outcomes. With few exceptions, most types of bullying victimizations and causes of serious injury were significantly associated with all four outcomes. Among the types of bullying victimization, the highest odds of suicide attempt and insomnia compared with subjects who were not bullied were among subjects who were left out of activities (i.e., were excluded from the group). Among the causes of injury, the highest AORs of loneliness, insomnia and suicidal ideation were among subjects who had received an injury due to an attack, compared with subjects who had never been seriously injured.

## 4. Discussion

The study aimed to measure the association of being bullied, fighting and injury, in terms of their frequency and nature, with negative psychological health outcome: loneliness, insomnia, suicidal ideation and suicide attempt in three Western Pacific Island countries. The study revealed that 9.3% felt lonely and 9.5% had insomnia, 27.6% had suicidal ideation, and 30.9% reported at least one suicide attempt in the last 12 months. A slightly higher prevalence of loneliness and insomnia, and a lower rate of suicidal ideation, was observed among Caribbean adolescents [29]. In contrast to the study, a lower rate of suicidal ideation and suicide attempt was observed among adolescents in Thailand, Peru, Malaysia and China [15,18,30,31,32]. One of the different findings of the study is that the prevalence of suicide attempt is higher than that of suicide ideation, which might indicate impulsive suicide attempts. The proportion of students being bullied, fighting, and sustaining injury was highest in the Solomon Islands. Similarly, the highest rates of loneliness, insomnia and suicide attempt were also observed in the Solomon Islands. These results might indicate a clustering of violence, psychological distress, and suicidal behavior. 

In the current study, being bullied 1 to 2 days and ≥3 days was significantly associated with all four psychological health outcomes, and the AORs increased with increased exposure to bullying compared to the non-bullied. Students who were in fight for once, 2 to 3 times or ≥4 times were more like to report loneliness, insomnia and suicidal behavior, and the AORs increased with the increased frequency of fighting compared to those who were not in a fight. Similar types of finding were observed between sustaining injury and all four outcomes. Although most of studies have not considered frequency of exposure, they report that the students who were victims of bullying and physical violence were usually found to report higher levels of suicidal ideation, suicide attempt and other poor psychological health [1,6,10,11,29]. 

In the current study, all types of bullying victimization and causes of injury had significant effects on loneliness, insomnia and suicidality except a few. The students who were left out of activities had the highest odds for insomnia and attempting suicide as compared to the non-bullied. In addition, adolescents who reported injury due to physical attack showed the highest odds of loneliness, insomnia and suicidal ideation among causes of injury as compared to those non-injured. The results showed that social exclusion and physical attack should be considered the most important factors affecting psychological health and suicidality.

### 4.1. Loneliness 

In the study, the participants who were bullied in the last 30 days prior to the survey, or were in fights or injured in the last 12 months were more likely to report loneliness, and the odds of loneliness usually increased with the degree of exposure to bullying, fighting and injury. A study comprising 19 low- and middle-income countries found that being bullied was statistically associated with the loneliness in most of the countries [6]. The students who were bullied were, in general, significantly more likely to report feeling lonely [6,10,11]. Loneliness, in turn, was associated with suicidal behavior, poor mental health and somatic disorders [1,7,33]. A population-based study also reports that the prevalence of suicide ideation increased with the degree of loneliness [33]. Thus, in this study, we have adjusted loneliness while computing the AORs of insomnia, suicidal ideation and suicide attempt. 

### 4.2. Insomnia 

In the study, we observed that adolescents who were bullied in the last 30 days, or were in fights or injured in the last 12 months were more likely to report insomnia. More importantly, odds of insomnia usually increased with the increased frequency of being bullied or fighting or injury. A study conducted among Finnish adolescents also showed that insomnia was most frequent among victims of bullying [8]. The association between being bullied and insomnia was found to be consistent with other studies conducted in low- and middle-income countries using GSHS data [6,10,11]. A study conducted among four South-East Asian countries observed that psychological distress, including loneliness and insomnia, was associated with higher odds of injury among adolescents [34]. On the other hand, insomnia itself was frequently found to be associated with suicidal behavior [20,21]. Thus, insomnia has been adjusted while calculating AORs of suicidal ideation and suicide attempt in this study.

### 4.3. Suicidal Behavior

A large number of studies indicated that students who were bullied or engaged in fighting within the past 12 months had higher odds of suicidal ideation or suicide attempt [13,15,16,18,31,35]. However, the above-mentioned studies did not assess the extent of exposure to fighting, bullying and the relative odds of suicidality. The current study found that the odds of suicidal ideation and suicide attempt among adolescents increased with the degree of exposure to bullying, fighting and injury. Although causality could not be proved due to the cross-sectional nature of data, the study showed a proportional association of violence and injury experience with suicidal behavior. A previous study showed that suicide attempters had significantly higher odds of being physically bullied as well as having a serious injury among school-attending Malawi adolescents [36]. 

In the present study, most types of bullying victimization had significant effects on suicidality. Among these, subjects who were left out of activities had the highest odds for attempting suicide. A study showed that adolescents who were threatened in school were at twice the risk of considering suicide [35]. Consistent to the study, all dimensions of victimization were associated with the three suicidal indicators (ideation, plan and attempt) in the island of O’ahu [37]. In the study, adolescents who reported injury due to physical attack showed the highest odds of suicidal ideation among causes of injury, compared to the non-injured. 

Bullying, fighting, injuries, psychological distress, and suicidality among adolescents might be clustered. Further prospective studies are needed to determine direction of causality. The results from the present study show an urgent need for the authorities concerned to develop and implement programs for the prevention and control of violence and injury to promote adolescent mental health and reduce suicidality.

### 4.4. Limitations of the Study 

The present study included data collected by the CDC using the standard questionnaire and methods, with a nationally representative sample size and an appropriate sampling method. However, several limitations exist. First, each outcome variable—loneliness, insomnia, suicidal ideation and suicide attempts—was assessed with a single item question instead of using a scale consisting of several items, which may result in some validity and reliability issues. Second, the question used to assess the nature of being bullied and causes of injury did not allow participants to report the experience of more than one form of bullying victimization or injury experience. Third, as a cross-sectional survey, causal inferences could not be made. Thus, a prospective study is essential to determine the direction of the causality. Fourth, since the responses were self-reported, methodological bias might exist. Fifth, sadness/hopelessness or depression was either not measured or not available in the publicly available dataset of the study countries. Sixth, some injuries might be due to non-suicidal self-injury or suicide attempt; however, this aspect could not be assessed in this study [38], because the question asked to measure the causes of injury did not provide specific information on it. 

### 4.5. Implication of the Study

Bullying, being in a fight and injury are serious public health problem in adolescents that require the attention of school administrations, educators, parents, school health and public health professionals. School health authorities should consider the effect of bullying victimization, fighting and injury on the psychological health of students. The Ministry of Education and the Ministry of Health should work in collaboration; policy makers should help school authorities to develop a system for safe and confidential recording, reporting and management of bullying and fighting at schools, and to create a safe and supportive environment for all. Integration of anti-bullying and injury prevention education into the curriculum might be more conducive to their prevention in the long run.

## 5. Conclusions 

The study revealed that being bullied, fighting, and injury, in terms of frequency and nature, were significantly associated with loneliness, insomnia and suicidal behavior, independent of substance abuse and parental support in adolescents in three Western Pacific Island countries. AORs of loneliness, insomnia, suicidal ideation and suicide attempt usually increased with increased exposure to bullying, fighting and injury compared to the non-exposed group. The study showed a proportional effect of exposure to violence and injury on the psychological health outcomes. Among the various types of bullying victimization, the highest AORs of insomnia and suicide attempt were found among subjects who were excluded from activities, compared to those non-bullied in last 12 months. Among the various causes of injury, the highest AORs of loneliness, insomnia, and suicidal ideation were found among subjects who were injured due to a physical attack, compared with subjects who were not injured in the last 12 months. Preventing violence and injury might be an important strategy for improving mental health and decreasing suicidal behavior among adolescents. 

## Figures and Tables

**Table 1:** Variable measurements.

Survey Questions	Coding	Variables
During the past 30 days, how many days were you bullied?	(0) None, (1) 1–2 days, (2) ≥3 days	Bullied
During the past 12 months, how many times were you in a physical fight?	(0) None, (1) 1 time, (2) 2–3 times, (3) ≥4 times	Fighting
During the past 12 months, how many times were you seriously injured? *	(0) None, (1) 1 time, (2) 2–3 times, (3) ≥4 times,	Injured
During the past 30 days, how many days did you smoke cigarettes?	(1) 1 or more times (0) none	Smoking
During the past 30 days, how many days did you have at least one drink containing alcohol?	(1) 1 or more times (0) none	Alcohol use
During your life, how many times have you used marijuana?	(1) 1 or more times (0) none	Marijuana use
During the past 30 days, how often did your parents or guardians know what you were doing with your free time? **	(1) Most of the time/always (0) Never/rarely/sometimes	Parental guidance
During the past 30 days, how often did your parents or guardians understand your problems and worries? **	(1) Most of the time/always (0) Never/rarely/sometimes	Parental understanding
During the past 30 days, how often did your parents or guardians go through your belongings without your approval? **	(1) Most of the time/always (0) Never/rarely/sometimes	Parental intrusion of privacy
During the past 12 months, how often have you felt lonely? **	(1) Most of the time/always (0) Never/rarely/sometimes	Loneliness
During the past 12 months, how often have you been so worried about something that you could not sleep at night? **	(1) Most of the time/always (0) Never/rarely/sometimes	Insomnia
During the past 12 months, did you ever seriously consider attempting suicide?	(1) Yes (0) No	Suicidal ideation
During the past 12 months, how many times did you attempt suicide?	(1) 1 or more times (0) None	Suicide attempt
During the past 30 days, how were you bullied most often?	(1–7) Bullying types (0) Not bullied	Types of bullying
During the past 12 months, what was the major cause of the most serious injury that happened to you?	(1–7) Injury causes (0) Not seriously injured	Causes of serious injury

* An injury is considered serious when you miss at least one full day of usual activities such as school, sports, or a job, or require treatment by a doctor or medical personnel; ** Most of the time/always is treated as “yes” and Never/rarely/sometimes as “no” in the analysis.

**Table 2:** Characteristics of the study population.

Variables	All (*n* = 4122)	Kiribati (*n* = 1582)	Solomon Islands (*n* = 1421)	Vanuatu (*n* = 1119)
Sex				
Male	1876 (45.5)	687 (43.4)	703 (49.5)	486 (43.4)
Female	2158 (52.4)	889 (56.2)	652 (45.9)	617 (55.1)
Missing	88 (2.1)	6 (0.4)	66 (4.6)	16 (1.4)
Age group				
≤14 years	2144 (52.0)	839 (53.0)	569 (40.0)	736 (65.8)
≥15 years	1893 (45.9)	725 (45.8)	798 (56.2)	370 (33.1)
Missing	85 (2.1)	18 (1.1)	54 (3.8)	13 (1.2)
Bullied				
None	1716 (41.6)	929 (58.7)	433 (30.5)	354 (31.6)
1–2 days	1251 (30.4)	361 (22.8)	477 (33.6)	413 (36.9)
≥3 days	767 (18.6)	148 (9.4)	368 (25.9)	251 (22.4)
Missing	388 (9.4)	144 (9.1)	143 (10.1)	101 (9.0)
Fighting				
None	2278 (55.3)	1024 (64.7)	668 (47.0)	586 (52.4)
1 time	924 (22.4)	336 (21.2)	316 (22.2)	272 (24.3)
2–3 times	524 (12.7)	168 (10.6)	205 (14.4)	151 (13.5)
≥4 times	374 (9.1)	52 (3.3)	219 (15.4)	103 (9.2)
Missing	22 (0.5)	2 (0.1)	13 (0.9)	7 (0.6)
Injured				
None	1222 (29.6)	525 (33.2)	348 (24.5)	349 (31.2)
1 time	1122 (27.2)	478 (30.2)	338 (23.8)	306 (27.3)
2–3 times	571 (13.9)	152 (9.6)	243 (17.1)	176 (15.7)
≥4 times	467 (11.3)	73 (4.6)	300 (21.1)	94 (8.4)
Missing	740 (18.0)	354 (22.4)	192 (13.5)	194 (17.3)
Smoking				
No	3036 (73.7)	1116 (70.5)	964 (67.8)	956 (85.4)
Yes	929 (22.5)	394 (24.9)	389 (27.4)	146 (13.0)
Missing	157 (3.8)	72 (4.6)	68 (4.8)	17 (1.5)
Alcohol use				
No	3022 (73.3)	1039 (65.7)	1001 (70.4)	982 (87.8)
Yes	883 (21.4)	478 (30.2)	304 (21.4)	101 (9.0)
Missing	217 (5.3)	65 (4.1)	116 (8.2)	36 (3.2)
Marijuana use				
No	3603 (87.4)	1487 (94.0)	1072 (75.4)	1044 (93.3)
Yes	347 (8.4)	63 (4.0)	242 (17.0)	42 (3.8)
Missing	172 (4.2)	32 (2.0)	107 (7.5)	33 (2.9)
Parental understanding				
No	3241 (78.6)	1330 (84.1)	1016 (71.5)	895 (80.0)
Yes	810 (19.7)	239 (15.1)	360 (25.3)	211 (18.9)
Missing	71 (1.7)	13 (0.8)	45 (3.2)	13 (1.2)
Parental guidance				
No	2956 (71.7)	1126 (71.2)	975 (68.6)	855 (76.4)
Yes	1105 (26.8)	432 (27.3)	415 (29.2)	258 (23.1)
Missing	61 (1.5)	24 (1.5)	31 (2.2)	6 (0.5)
Parental intrusion of privacy				
No	1806 (43.8)	553 (35.0)	808 (56.9)	445 (39.8)
Yes	2225 (54.0)	1009 (63.8)	549 (38.6)	667 (59.6)
Missing	91 (2.2)	20 (1.3)	64 (4.5)	7 (0.6)
Loneliness				
No	3706 (89.9)	1483 (93.7)	1188 (83.6)	1035 (92.5)
Yes	384 (9.3)	95 (6.0)	210 (14.8)	79 (7.1)
Missing	32 (0.8)	4 (0.3)	23 (1.6)	5 (0.4)
Insomnia				
No	3686 (89.4)	1428 (90.3)	1200 (84.4)	1058 (94.5)
Yes	390 (9.5)	148 (9.4)	181 (12.7)	61 (5.5)
Missing	46 (1.1)	6 (0.4)	40 (2.8)	0 (0.0)
Suicidal ideation				
No	2878 (69.8)	1003 (63.4)	972 (68.4)	903 (80.7)
Yes	1136 (27.6)	532 (33.6)	412 (29.0)	192 (17.2)
Missing	108 (2.6)	47 (3.0)	37 (2.6)	24 (2.1)
Suicide Attempt				
None	2784 (67.5)	1069 (67.6)	853 (60.0)	862 (77.0)
≥1 time	1272 (30.9)	491 (31.0)	524 (36.9)	257 (23.0)
Missing	66 (1.6)	22 (1.4)	44 (3.1)	0 (0.0)

**Table 3:** Type of bullying victimization and causes of serious injury.

Variables	Number	Percentage
Type of bullying		
Not bullied	2110	51.2
Kicked/pushed/shoved	414	10.0
Made fun of due to their race	236	5.7
Made fun of due to their religion	175	4.2
Made fun of regarding their sex	240	5.8
Left out of activities	80	1.9
Made fun of their body	168	4.1
Other types	322	7.8
Missing	377	9.1
Causes of serious injury		
Not seriously injured	2083	50.5
Motor vehicle accident	147	3.6
I fell down	468	11.4
Something fell on me or hit me	285	6.9
I was attacked	178	4.3
I was in a fire	127	3.1
Breathed something bad	63	1.5
Something else	317	7.7
Missing	454	11.0

**Table 4:** Association of being bullied, in fight, and injury with psychological health outcomes.

Variables	Unadjusted OR			
	Loneliness	Insomnia	Suicidal Ideation	Suicide Attempt
Bullying				
None	1	1	1	1
1–2 days	1.81 (1.37–2.39) ^a^	1.80 (1.36–2.38) ^a^	1.87 (1.58–2.21) ^a^	2.52 (2.13–2.99) ^a^
≥3 days	3.17 (2.38–4.20) ^a^	3.49 (2.64–4.62) ^a^	2.29 (1.89–2.77) ^a^	4.21 (3.48–5.08) ^a^
Fighting				
None	1	1	1	1
1 time	2.00 (1.53–2.62) ^a^	1.70 (1.30–2.21) ^a^	1.63 (1.37–1.93) ^a^	1.95 (1.65–2.31) ^a^
2–3 times	2.13 (1.55–2.93) ^a^	1.90 (1.40–2.60) ^a^	1.64 (1.33–2.03) ^a^	2.68 (2.19–3.27) ^a^
≥4 times	4.18 (3.08–5.67) ^a^	3.19 (2.33–4.36) ^a^	2.30 (1.82–2.91) ^a^	4.46 (3.54–5.61) ^a^
Injured				
None	1	1	1	1
1 time	2.66 (1.88–3.74) ^a^	2.11 (1.49–2.99) ^a^	1.83 (1.51–2.21) ^a^	2.55 (2.10–3.11) ^a^
2–3 times	2.66 (1.80–3.94) ^a^	3.31 (2.28–4.80) ^a^	2.04 (1.62–2.56) ^a^	3.61 (2.88–4.53) ^a^
≥4 times	5.63 (3.90–8.12) ^a^	5.20 (3.61–7.49) ^a^	2.62 (2.06–3.32) ^a^	5.49 (4.32-6.97) ^a^
Background variables				
Male sex	0.96 (0.78–1.19)	1.00 (0.80–1.23)	0.94 (0.82–1.08)	0.90 (0.78–1.03)
≤14 years	0.70 (0.56–0.87) ^b^	0.77 (0.62–0.95) ^c^	0.84 (0.73–0.97) ^c^	0.77 (0.68–0.89)
Smoking	2.20 (1.75–2.76) ^a^	2.30 (1.84–2.88) ^a^	2.59 (2.22–3.04) ^a^	3.05 (2.60–3.56) ^a^
Alcohol use	1.99 (1.58–2.52) ^a^	2.12 (1.68–2.67) ^a^	2.45 (2.08–2.88) ^a^	2.83 (2.42–3.32) ^a^
Marijuana use	4.05 (3.08–5.34) ^a^	2.73 (2.03–3.68)	2.44 (1.94–3.08) ^a^	3.75 (2.98–7.72)
Parental understanding	1.70 (1.34–2.16) ^a^	1.25 (0.97–1.61)	0.92 (0.77–1.09)	1.19 (1.00–1.40)
Parental guidance	1.18 (0.93–1.48)	1.26 (1.00–1.59) ^c^	0.71 (0.60–8.37) ^a^	0.91 (0.78–1.06)
Parental interference	1.50 (1.21–1.86) ^a^	0.62 (0.50–0.77) ^a^	1.16 (1.00–1.33) ^c^	0.68 (0.59–0.78)
Country				
Kiribati	0.83 (0.61–1.14)	1.79 (1.32–2.44) ^a^	2.49 (2.06–3.01) ^a^	1.54 (1.29–1.83) ^a^
Solomon Islands	2.31 (1.76–3.03)	2.61 (1.93–3.53) ^a^	1.99 (1.64–2.42) ^a^	2.06 (1.72–2.45) ^a^
Vanuatu	1	1	1	1
Adjusted OR *				
Bullied				
None	1	1	1	1
1–2 days	1.42 (1.03–1.94) ^c^	1.96 (1.40–2.73) ^a^	2.22 (1.81–2.73) ^a^	2.03 (1.62–2.55) ^a^
≥3 days	1.76 (1.24–2.50) ^b^	2.48 (1.71–3.60) ^a^	2.37 (1.84–3.04) ^a^	2.43 (1.84–3.20) ^a^
Fighting				
None	1	1	1	1
1 time	1.78 (1.31–2.43) ^a^	1.44 (1.04–1.99) ^c^	1.55 (1.26–1.90) ^a^	1.46 (1.15–1.83) ^b^
2–3 times	1.56 (1.06–2.28) ^c^	1.72 (1.18–2.51) ^b^	1.59 (1.24–2.05) ^a^	1.98 (1.49–2.62) ^a^
≥4 times	2.62 (1.78–3.85) ^a^	2.29 (1.51–3.47) ^a^	1.97 (1.45–2.68) ^a^	2.46 (1.75–3.47) ^a^
Injured				
None	1	1	1	1
1 time	2.04 (1.40–2.98) ^a^	1.90 (1.27–2.84) ^b^	1.58 (1.27–1.98) ^a^	1.80 (1.39–2.32) ^a^
2–3 times	2.09 (1.36–3.22) ^b^	2.89 (1.86–4.48) ^a^	1.75 (1.34–2.30) ^a^	2.49 (1.84–3.38) ^a^
≥4 times	3.41 (2.20–5.28) ^a^	2.75 (1.69–4.47) ^a^	2.32 (1.71–3.17) ^a^	2.51 (1.77–3.58) ^a^

^a^ *p*-value < 0.001, ^b^ *p*-value < 0.01, ^c^ *p*-value < 0.05; * Adjusted for age, sex, country, smoking, alcohol use, marijuana use, parental understanding, parental knowledge of free time use, parental interference and primary sampling unit, and the ORs of insomnia were also adjusted with loneliness, those of suicidal ideation with loneliness and insomnia, and those of suicide attempt with loneliness, insomnia and suicidal ideation.

**Table 5:** Association of bullying types and injury causes with psychological health outcomes.

Variables	Adjusted OR *			
	Loneliness	Insomnia	Suicidal Ideation	Suicide Attempt
*Type of bullying*				
Not bullied	1	1	1	1
Kicked/pushed	1.46 (0.97–2.19)	1.75 (1.15–2.67) ^b^	1.97 (1.49–2.59) ^a^	1.62 (1.18–2.21) ^b^
Made fun of due to their race	1.4 2 (0.85–2.39)	2.66 (1.63–4.34) ^a^	2.03 (1.41–2.91) ^a^	2.10 (1.41–3.14) ^a^
Made fun of due to their religion	1.25 (0.70–2.23)	1.88 (1.06–3.30) ^c^	1.75 (1.19–2.55) ^b^	2.59 (1.70–3.96) ^a^
Made fun of regarding their sex	1.97 (1.23–3.14) ^b^	1.23 (0.71–2.13)	2.18 (1.57–3.04) ^a^	1.76 (1.21–2.56) ^b^
Left out of activities	1.48 (0.69–3.19)	4.20 (2.14–8.26) ^a^	1.91 (1.06–3.43) ^c^	3.57 (1.90–6.71) ^a^
Made fun of their body	1.30 (0.70–2.42)	2.04 (1.12–3.68) ^c^	2.91 (1.96–4.32) ^a^	1.46 (0.91–2.34)
Other types	1.17 (0.74–1.87)	1.41 (0.85–2.34)	1.67 (1.21–2.31) ^b^	1.58 (1.11–2.24) ^c^
*Causes of serious injury*				
Not seriously injured	1	1	1	1
Involved in motor vehicle accident	1.97 (1.02–3.81) ^c^	3.07 (1.68–5.62) ^a^	1.96 (1.24–3.09) ^b^	2.22 (1.32–3.74) ^b^
I fell	1.89 (1.26–2.82) ^b^	1.85 (1.20–2.85) ^b^	1.51 (1.14–1.99) ^b^	1.66 (1.22–2.25) ^b^
Something fell on me or hit me	2.38 (1.50–3.78) ^a^	2.36 (1.44–3.86) ^b^	1.90 (1.34–2.69) ^a^	2.69 (1.83–3.95) ^a^
I was attacked	4.51 (2.79–7.28) ^a^	3.90 (2.40–6.34) ^a^	2.38 (1.61–3.54) ^a^	2.40 (1.52–3.80) ^a^
I was in a fire or too near a flame	2.26 (1.17–4.37) ^c^	1.74 (0.88–3.44)	1.81 (1.15–2.84) ^b^	1.99 (1.18–3.35) ^c^
I breathed or swallowed something bad for me	3.87 (1.83–8.16) ^a^	1.33 (0.49–3.59)	2.30 (1.22–4.34) ^b^	1.65 (0.81–3.38)
Something else caused my injury	2.12 (1.35–3.34) ^b^	1.66 (1.02–2.69) ^c^	1.33 (0.98–1.81)	1.45 (1.02–2.06) ^c^

^a^ *p*-value < 0.001, ^b^ *p*-value < 0.01, ^c^ *p*-value < 0.05; * Adjusted for age, sex, country, smoking, alcohol use, marijuana use, parental understanding, parental knowledge of free time use, parental interference, and primary sampling unit, and the ORs of insomnia was also adjusted with loneliness; those of suicidal ideation with loneliness and insomnia, and those of suicide attempt with loneliness, insomnia and suicidal ideation.
